# A New Secondary Pollen Presentation Mechanism from a Wild Ginger (*Zingiber densissimum*) and Its Functional Roles in Pollination Process

**DOI:** 10.1371/journal.pone.0143812

**Published:** 2015-12-04

**Authors:** Yong-Li Fan, W. John Kress, Qing-Jun Li

**Affiliations:** 1 Key Laboratory of Tropical Forest Ecology, Xishuangbanna Tropical Botanical Garden, The Chinese Academy of Sciences, Menglun Town, Mengla County, Yunnan, China; 2 University of Chinese Academy of Sciences, Beijing, China; 3 China Forest Exploration and Design Institute of Kunming, Kunming, China; 4 Department of Botany, National Museum of Natural History, Smithsonian Institution, Washington, DC, United States of America; University of Cologne, GERMANY

## Abstract

**Background and Aims:**

Secondary pollen presentation (SPP), a floral mechanism of reproductive adaptation, has been described for more than 200 years, with nine types SPP recorded. However, few studies have been done experimentally to link the floral mechanism of SPP to its functional roles in pollination process. This study aims to describe a new SPP mechanism from a wild ginger (*Zingiber densissimum*, Zingiberaceae) and explore how the pollen arrangement of SPP affects pollen removal during the interaction with different pollinators.

**Methodology/Principal Findings:**

Field observations and experiments revealed that flowers lasted for less than one day. The breeding system was partially self-incompatible. Two bee species, *Macropis hedini* (which carried pollen dorsally) and *Amegilla zonata* (which carried pollen ventrally) were the primary pollinators. About a third of pollen grains were relocated from the anther to the labellum staminode of flowers through the adherence of aggregated pollen chains, while other grains were presented on the anther. In a single visit, each bee species removed pollen grains from both the labellum staminode and the anther. *Macropis hedini* was more effective than *Amegilla zonata*.

**Conclusions/Significance:**

Our study describes a new SPP mechanism in angiosperms. The new SPP mode enables pollen grains presented on the anther and the labellum staminode simultaneously via the adherence of aggregated pollen chains, thus promoting pollen to be taken away by different pollinators. This SPP mechanism plays a key role during pollen removal and may have evolved under the pressure to improve male fitness.

## Introduction

Most flowers are cosexual, garnering fitness via both receipt of pollen (female function) and dispersal of pollen (male function). For many animal-pollinated flowers, the receipt of pollen can be saturated by only a few visits of pollinators, whereas siring success by pollen dispersal generally continues to increase through numerous visits by pollinators [1]. Therefore, selective pressure on floral mechanisms is stronger via male function than female function. Flowers are sometimes even thought to be primarily for the export of pollen rather than for the receipt of pollen [1, 2]. Under selection to improve male function, the pollen presentation mechanisms that promote pollen transfer will be favored [3–6].

There are two fundamental pollen presentation strategies to ensure maximum siring success in angiosperms: gradual pollen presentation strategy and simultaneous pollen presentation strategy. Either simultaneous or gradual pollen presentation has both benefit and cost, and can be adaptive depending on varied circumstances. In gradual pollen presentation strategy, flowers present pollen to pollinators step by step (e.g., the style growing through the anther tube and exposing gradually the pollen in some species of Campanulaceae and Compositae, and the anther opening with a pore in Melastomataceae and Ericaceae) [5, 7–12]. Gradual pollen presentation strategy promotes the efficiency of pollen deposition on stigmas. But restriction on pollen removal may increase (1) the risk of leaving pollen on anthers even after flower withers, and (2) the cost of pollen removal (e.g., in pollinator rewards and floral longevity sustenance) [13–16]. Therefore, in flowers with gradual pollen presentation, benefit from male function is primarily achieved by enhancing the efficiency of pollen deposition. In simultaneous pollen presentation strategy, flowers simultaneously present all the pollen to pollinators via (1) all anthers maturing and dehiscing simultaneously (e.g., in Myrtaceace) [17], (2) aggregated pollen presentation (e.g., pollinia in orchids) [18], or (3) some special trigger mechanisms (e.g., explosive pollen presentation in Marantaceae) [19, 20]. Simultaneous presentation strategy promotes the amount of pollen export in a single visit by a pollinator, the benefits from which far overweigh the costs induced by diminishing returns of pollen deposition and the decrease in pollen deposition efficiency [8, 21, 22]. Therefore, in flowers with simultaneous pollen presentation, benefit from male function is primarily achieved by promoting the amount of pollen export rather than by enhancing pollen deposition efficiency.

Secondary pollen presentation (SPP) is a floral mechanism of pollination adaptation in which pollen is presented on floral structures (the 2^nd^ presenter) other than anthers [23, 24]. For example, when pollen is moved by the flower from inside anthers to hairs on the style where pollinators pick it up, an SPP mode is formed. This is what happens in *Campanula*, and was first described by Sprengel in 1793. Since then, SPP has been recorded from 16 families, and nine modes of SPP have been distinguished based on the differences in where and how pollen grains are loaded [10, 24]. SPP shows simultaneous pollen presentation strategy in some species (e.g., in Myrtaceae, Marantaceae and Cannaceae) [24], but gradual pollen presentation strategy in other species (e.g., in Lobeliaceae and Compositae) [9, 25]. Whether simultaneous or gradual, SPP is postulated to promote pollen dispersal in different manners. In addition, SPP may lengthen the male phase and increase the efficiency of pollen transfer [24]. A cost of SPP is sometimes considered, involving sexual interference between pollen grains and stigmas [24, 26, 27]. The multiple origins of SPP in angiosperms also suggest SPP is adaptive [10]. However, the functional and evolutionary assumptions made regarding adaptive values of SPP are based mainly on morphological descriptions, and few studies have been done experimentally to evaluate how flowers achieve benefit through the interaction between SPP and pollinator’s behaviours during pollination [27].

Gingers (Zingiberales) are ancestrally pollinated by animals [28]. In Zingiberales, some extremely specialized pollination systems and unique pollination mechanisms are found, such as flexistyly [29], deceit pollination [30] and trigger mechanism of pollen presentation [19, 31], indicating pollinators have long been of crucial importance during the sexual reproduction. SPP mechanism has been reported in two ginger families (Marantaceae and Cannaceae), in which SPP plays an important role in the interaction with pollinators [10, 32, 33]. However, there is no report in Zingiberaceae. Here we described a novel SPP mechanism in nature from a wild ginger (*Zingiber densissimum* S.Q.Tong & Y.M.Xia) of Zingberaceae. Our preliminary field observation indicated that pollen in this species was not only exposed in the anther but also in other structures such as the labellum. We are therefore intrigued to explore the mechanisms of reproductive adaptation in this species. We address the following issues: (1) What are the features of pollination ecology in *Z*. *densissimum*? (2) How is SPP achieved in *Z*. *densissimum*? (3) How does SPP affect pollen export during the pollination process by different pollinators?

## Materials and Methods

### Study system and area

*Zingiber densissimum* is a perennial herb distributed in southwestern Yunnan and Thailand. Pseudostems are 40–70 cm tall. Inflorescences arise from rhizomes, with peduncles attached below ground level. Bracts are red apically; bracteoles are silvery villous. Flowers are generally white, with one central lobe and two lateral lobes. Four stamenoids (sterile stamens) are fused to form a three-lobed petaloid labellum staminode, and only one stamen is fertile. The anther is light yellow, with a yellow connective extending at the top, forming a tail-like anther appendage. The filiform style passes through the anther, and is fixed by the two pollen sacs [34].

This study was done in Lincang, Yunnan province, southwest of China (23°35′N, 100°04′ E; altitude 1890 m), in the flowering seasons of 2011 and 2012. The study population grew in a pine forest. Permission was got from the owner of the land to conduct the study on this site. No endangered or protected species were involved in this study.

### Floral biology, secondary pollen presentation mechanism and breeding system

In 2011, about 30 plant individuals were randomly selected for documenting phenology and floral morphology. The number of inflorescences per individual, the number of flowers per inflorescence, floral intervals per inflorescence, floral longevity and the length of corolla tube for each flower were measured. To assess nectar secretion, single flowers from 30 individuals were bagged with fine-mesh nylon bags before anthesis to exclude insects. At noon, nectar volume and sugar concentrations were measured using micro-capillaries (5 and 10 μL) and a refractometer (Eclipse 45–81; Bellingham and Stanley Ltd, Tunbridge Wells, Kent, UK), respectively. In addition, single flower buds from 30 individuals were randomly collected and fixed in 70% ethanol separately for pollen and ovule counts. The pollen: ovule (P/O) ratio of each flower was then calculated via the number of pollen grains divided by the number of ovules.

To examine the percentages of pollen grains presented on the anther and the labellum staminode, single flowers from 30 individuals were bagged with fine-mesh nylon bags before anthesis to exclude insects in 2012. The next day, the anther and the labellum staminode were carefully removed from each fresh flower and fixed in ethanol separately for pollen counts.

Pollen chain is a key trait for the occurrence of this novel SPP mechanism in *Z*. *densissimum*. To explore why pollen grains are aggregated together through the adherence of pollen surface to form pollen chains, pollen wall structures were explored. As SEM photography provided little information about pollen wall structure according to both previous researches [35, 36] and our preliminary study, a new method was developed for the exploration of pollen wall structures in *Z*. *densissimum* in this study. Fresh pollen grains were collected from field, fixed in FAA solution and taken back to the lab. Pollen grains were then put on the glass slide with FAA solution, stained with aniline blue and observed under a light microscope. After pollen grains got dried on the glass slide, pure water was then added on the glass slide. The different layers of pollen wall would gradually separate with each other during water swelling because of the difference in swelling ability of each layer. The pollen grain was then put on a fluorescence microscope to observe the structures of each layer. The layers that contain callose would reflect fluorescence.

To determine the breeding system, single flowers from more than 120 individuals were randomly selected in 2011. Visitors were excluded using nylon mesh bags. Plants were evenly and randomly allocated to four pollination treatments: (1) flowers were emasculated before anthesis and bagged, (2) flowers were left intact and bagged, (3) flowers were hand pollinated using pollen grains from the same flower and bagged, and (4) flowers were emasculated before anthesis, hand pollinated using pollen grains from flowers of other individuals and bagged. Treatments (3) and (4) were repeated in the 2012 flowering season. Fruits were collected 30 days after manipulation, and fruit set and mean seed number were calculated. Flowers that did not set fruit were not taken into account for mean seed number. To examine the difference in fruit set, a logistic model was used. To test for differences in seed number per fruit between hand self-pollination and hand cross-pollination, a two-way ANOVA was used with year and treatment as fixed factors. Year, treatment and year × treatment terms were considered in both the seed number and the fruit set analyses.

### Pollinator observation

Flower visitors were observed on sunny days continuously from 0900 h to 1700 h for three days in peak flowering seasons of 2011 and 2012. Pollinator species, pollinator behaviours and the total number of pollinator visits were recorded. One sample per observed pollinator species was caught for species identification.

### Pollination efficiency and pollen removal

To explore the adaptive significance of this new SPP, pollination efficiency of each pollinator species was estimated by both the seed production and the amount of pollen remaining after a single visit of a pollinator to a virgin flower. In 2012, flowers were covered with fine-mesh nylon bags before anthesis to exclude insects. The next day, each freshly opened flower was exposed for a single pollinator visit. Upon visitation, the identity of the visitor was recorded, and the anther and the labellum staminode were carefully removed from the flower and fixed in ethanol separately so as to later count pollen remaining on each floral structure. The flowers were then tagged and covered again with the nylon bags until the flowers closed. Fruits were collected 30 days after visitation, and seeds were counted. For the flowers that did not set seeds, the seed number was zero. About 40 flowers from a total of 40 individuals were treated for each bee species to evaluate seed production. A subset of 30 flowers visited by each bee species were randomly selected to evaluate the amount of pollen grains remaining on the donor’s structure. Control flowers were also arrayed. For control flowers, the anther and the labellum staminode from 30 virgin flowers were collected for pollen counts, and then flowers were tagged and re-bagged for later fruit collection. During the treatment process, pollinator visitation was not allowed. In this experiment, the total number of remaining pollen grains on each flower was calculated as the number of grains on the anther plus the number of grain on the labellum staminode.

The difference in seed production among the control group, the ventral pollination group and the dorsal pollination group was analyzed using a Kruskal-Wallis test followed by multiple comparisons, given the non-normality of the data. The differences among the three groups in the number of total number of remaining pollen (TP), the number of pollen on the anther (AP), and the number of pollen on the labellum staminode (LP) were examined using one-way ANOVA followed by a Tukey multiple comparisons, respectively. For the analysis of the difference in LP among groups, the data were transformed as 1/*Y* to improve normality.

In this study, all statistical analyses were done in R 3.02, and data were expressed as the mean ± standard error.

## Results

### Floral biology, secondary pollen presentation mechanism and breeding system

Peak flowering was in mid-August. Plant typically had one to four inflorescences and 11 to 20 flowers per inflorescence, with one flower open on each inflorescence at a time. Flowers persisted for less than a day, opening in the morning (about 0700 h) and closing in the late evening (about 2100 h). Pollen sacs dehisced in the morning after the flowers opened. Flowers provided nectar to pollinators. The P/O ratio was 417.7 ± 11.7 (*n* = 30; Table 1). The more information about floral characteristics was shown in Table 1.

**Table 1:** Floral characteristics (mean ± s.e., with sample size in brackets) of *Zingiber densissimum*.

Floral characters	unit	value
Flowers per inflorescence		16.1 ± 1.0 (10)
Flowering intervals	day	1.15 ± 0.07 (61)
Floral longevity	day	1 (50)
Corolla tube length	cm	4.94 ± 0.05 (28)
Nectar volume	μL	9.1 ± 0.3 (30)
Sucrose concentration	**%**	37.7± 0.3 (30)
Pollen grains		9163 ± 280 (30)
Ovules		22.2 ± 0.7 (30)
P/O ratio		417.7 ± 11.7 (30)

**Fig 1:**
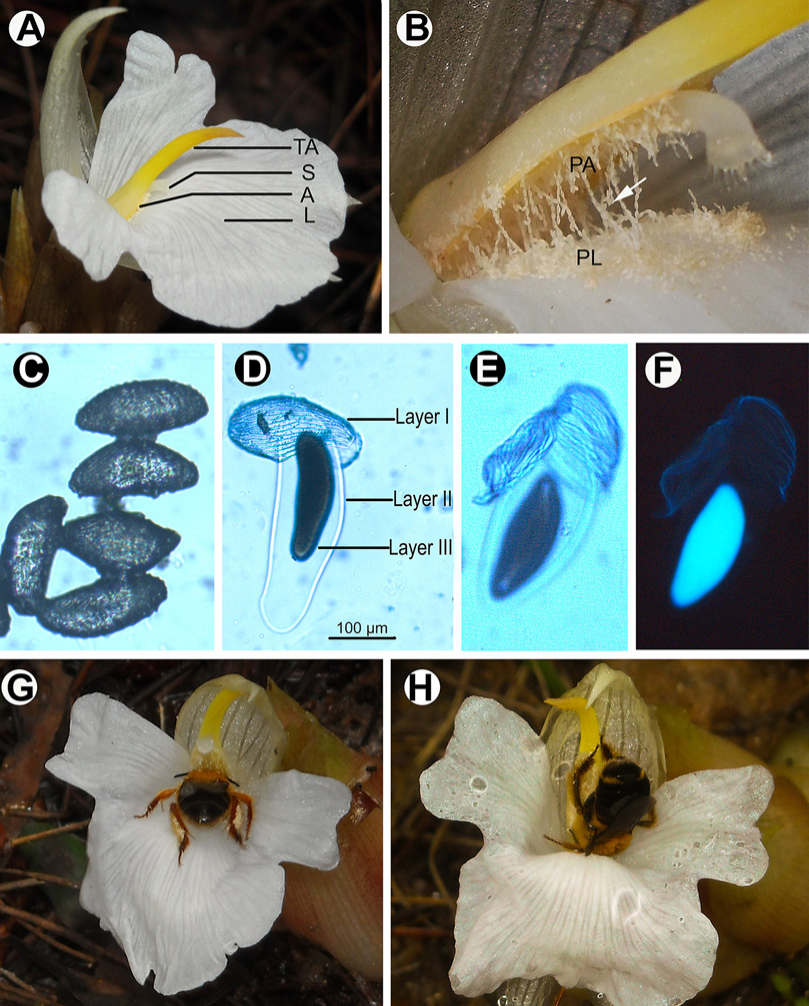
Zingiber densissimum flower, pollen and pollinators. (A): The floral stucture of *Zingiber densissimum*: TA, tail-like anther crest; S, stigma; A: anther; L: labellum staminode. (B): A fresh flower with the anther appendage manually pushed up to show the secondary pollen presentation mechanism: PA, pollen on the anther; PL, pollen on the labellum staminode; the arrow shows the aggregated pollen chains. (C)-(F): pollen conditions under different treatments, images are to the same scale. (C): Pollen grains that were fixed in FAA solution and observed under a light microscope after stained with aniline blue, with all layers of pollen wall sticking together. (D): A pollen grain observed under a light microscope after water swelling, with all three layers of pollen wall separated. (E): A pollen grain stained with aniline blue and observed under a light microscope after water swelling. (F): The same pollen grain as (E) observed under a fluorescence microscope, with only the layer III reflecting fluorescence. (G): A flower dorsally pollinated by a bee of *Macropis hedini*. (H): A flower ventrally pollinated by a bee of *Amegilla zonata*, with a leg of the bee touching the stigma.

The sole fertile anther is clsoe to the labellum staminode (Fig 1A). As pollen sacs dehisce, pollen grains were exposed outside of the pollen sacs, and some of the pollen grains directly contact with the labellum staminode, because of the close proximity of the anther to the labellum staminode (Fig 1A). Pollen grains are aggregated together to form pollen grain chains (Fig 1B). The connection of pollen grains in pollen chain is a temporary bond through the adherence of outer surface of pollen. The pollen chain may be broken under external force to form separated pollen grains, and the latter may rearrange to form new pollen chains when they met other pollen grains, according to our field observation. Numerous pollen grains in chains adhere to the labellum staminode (Fig 1B). In the natural condition, the average number of pollen grains left on the anther was 5394 ± 131 (*n* = 30), while the number of pollen grains presented on the labellum staminode was 3388 ± 99 (*n* = 30), i.e., 38% of the total pollen grains.

All layers of pollen wall were adhered together when pollen was fixed in FAA solution or kept dry naturally (Fig 1C). After pure water was added, three layers (layer I, II and III) of the pollen wall were separated, with the outer layer (layer I) showing a structure of spiro-striate sculpturing in a light microscope (Fig 1D), the middle layer (layer II) showing a membrane structure capable of water absorbing (Fig 1D), and the inner layer (layer III) capable of reflecting fluorescence (Fig 1E and 1F).

**Fig 2:**
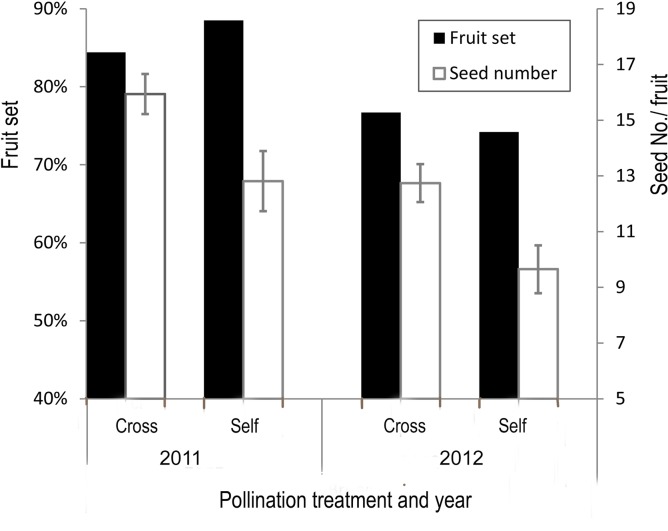
Breeding system of *Zingiber densissimum*, showing the difference between hand cross-pollinated flowers and hand self-pollinated flowers in fruit set and seed number in 2011 and 2012, respectively.

Flowers that were emasculated and bagged before anthesis did not set fruits (*n* = 34), indicating no apomixis. Unmanipulated bagged flowers did not produce seed (*n* = 50), suggesting no autonomous self-pollination. Hand self-pollinated flowers set as many fruits as hand cross-pollinated flowers (logistic model, Fig 2, Table 2). The seed number of hand cross-pollinated flowers was significantly higher than that of hand self-pollinated flowers, while the difference of seed production between years is not significant (two-way ANOVA, Fig 2, Table 2). These results indicate the breeding system of *Z*. *densissimum* is partially self-incompatible.

**Table 2:** Results of an analysis of deviance (χ^2^) and a two-way ANOVA (F) examining the differences in fruit set and seed number between hand self-pollination and hand cross-pollination in *Zingiber densissimum*.

Source	Fruit set			Seed number		
	DF	χ^2^	p	DF	F	p
year	1	2.681	0.1016	1	13.42	0.0004
treatment	1	0.037	0.8479	1	12.37	0.0006
Year × treatment	1	0.300	0.5837	1	0.001	0.9790

### Pollinator observation

Two bee species, *Macropis hedini* (Melittidae) and *Amegilla zonata* (Apidae), commonly visited flowers of *Z*. *densissimum*. To get at nectar, they need push up the anther, an obstacle located on the entrance of labellum staminode (Fig 1A). After landing on the labellum staminode, the two bees behaved differently during probing for nectar. *Macropis hedini* pushed up the anther appendage from the labellum staminode with its head, and then directly stepped forward to take the nectar, with the venter of the body contact the labellum staminode and the dorsum touching the stigma and the anther, so it pollinated flowers dorsally and took the pollen through legs and the venter of the body (Fig 1G). In contrast, *Amegilla zonata* stepped forward to hold the anther and the anther appendage with legs at first, and then rolled its body down and pushed up the anther appendage to take the nectar, with the dorsum contact the labellum staminode and the venter touching the anther, so it pollinated flowers ventrally and took the pollen on the labellum through the head, legs and the dorsum of the body (Fig 1H and S1 Fig). During a single nectar foraging process, both pollinators need hold and push the tail-like anther appendage with forelegs to get the nectar (Fig 1H), and its head and forelegs contacted the stigma under the anther appendage. The pollen from the labellum of the previous flower would be deposited on the stigma. After retreating from floral tube, bees, streaked with pollen, groomed off some pollen grains from the body. During this process, some pollen grains were lost and others rearranged on the pollinator body. In 2011, a total of 76 pollinator visits were recorded during the three days of observation, with 52 visits by the ventral pollinator (*A*. *zonata*) and 24 visits by the dorsal pollinator (*M*. *hedini*), while in 2012, a total of 68 visits were recorded, with 23 visits by the ventral pollinator (*A*. *zonata*) and 45 visits by the dorsal pollinator (*M*. *hedini*). The result shows the frequency of two bee species visitation fluctuates between the two years (Chi-squared test, *χ* ^*2*^ = 15.85, *P* < 0.001).

### Pollination efficiency and pollen removal

**Fig 3:**
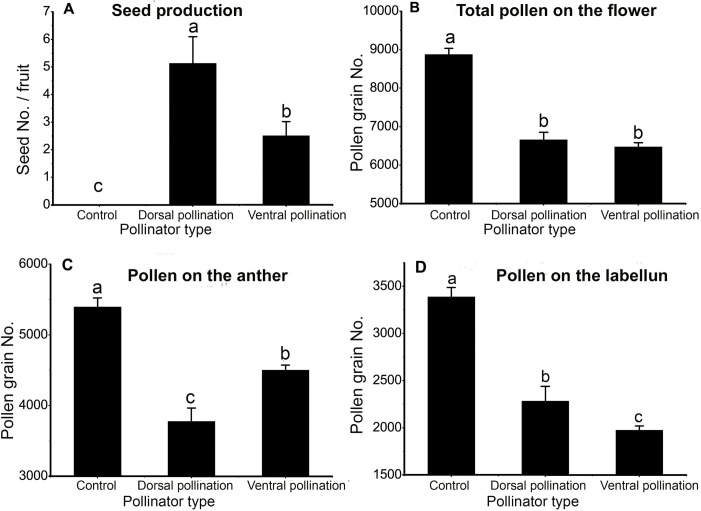
Pollination efficiency and pollen left after a single visit by different pollinator species. Pollinator type: Control, flowers were not visited by pollinators; Dorsal pollination, flowers were dorsally pollinated by bees of *Macropis hedini*; Ventral pollination, flowers were ventrally pollinated by bees of *Amegilla zonata*. Values with the different letters indicate which comparisons are significantly different (P < 0.05) using Tukey post-hoc multiple comparisons.

Flowers visited once by the dorsal pollinator (*M*. *hedini*) produced significantly more seeds than flowers visited once by the ventral pollinator (*A*. *zonata*), and both groups produced significantly more seeds than the control group (Kruskal-Wallis test, *χ* ^*2*^ = 34.53, *P* < 0.001; Fig 3A). This indicates both bees were able to contribute to the plant fitness after one visit enabling pollen removal and seed production; the dorsal pollinator (*M*. *hedini*) has higher pollination effectiveness than the ventral pollinator (*A*. *zonata*) in a single visit. The total number of remaining pollen grains was significantly lower when a flower was visited than for controls, while there was no significant difference between the two pollinators (one-way ANOVA, *F* = 63.25, *P* < 0.001; Fig 3B), showing the pollen removal efficiencies are similar between dorsal pollinators (*M*. *hedini*) and ventral pollinators (*A*. *zonata*). The amount of pollen grains left on the anther was significantly lower in flowers visited by dorsal pollinators (*M*. *hedini*) than in flowers visited by ventral pollinators (*A*. *zonata*) (one-way ANOVA, *F* = 33.6, *P* < 0.001; Fig 3C), indicating dorsal pollinators (*M*. *hedini*) remove more pollen grains from the anther than ventral pollinators(*A*. *zonata*) during a single visit. However, the number of pollen grains left on the labellum staminode was significant lower in flowers visited by ventral pollinators (*A*. *zonata*) than flowers visited by dorsal pollinators (*M*. *hedini*), and the number of pollen grains left on the labellum staminode in these two bee-visited flower groups was significantly lower than that in the control group (one-way ANOVA, *F* = 63.17, *P* < 0.001; Fig 3D). The result indicates (1) both bee species remove pollen grains from the labellum staminode, and (2) ventral pollinators (*A*. *zonata*) remove more pollen grains from the labellum staminode than dorsal pollinators (*M*. *hedini*) in a single visit.

## Discussion

Our study describes a new mode of secondary pollen presentation (SPP) in nature from *Zingiber densissimum*, in which about a third of pollen grains are presented on the labellum staminode (the 2^nd^ presenter) through the adherence of aggregated pollen chains while other grains are simultaneously presented on the anther (Fig 1B). This is the first report on the occurrence of pollen chains associated with SPP in angiosperms and the first record of SPP in Zingiberaceae. We found this new SPP mode promotes the capacity of different pollinators to remove pollen (Fig 3D). As a simultaneous pollen presentation strategy, this new SPP mode is probably evolved under selection via male function.

### Floral biology

Each individual of *Z*. *densissimum* generally has one flower open per inflorescence each day, with floral longevity less than one day. No autonomous selfing was found, and flowers were partially self-incompatible (Fig 2, Table 2). The P/O ratio of 417.7 ± 11.7 places the species in the category of facultative xenogamy [37]. All these results indicate Z. *densissimum* is a xenogamous species. Although autonomous selfing mechanisms have been reported from a few ginger species [38–40], xenogamy is thought to be the dominant breeding system among Zingiberales [28]. *Zingiber densissimum* has showy zygomorphous flowers, nectar rewards and the labellum staminode as the landing platform, flowering at daytime, which show the pollination syndrome of bees [23]. This matches the bee pollination we observed in the field, suggesting a stable plant-bee interaction sustains in *Z*. *densissimum*.

### A new arrangement of secondary pollen presentation

Previous records have led to the recognition of nine types of SPP from 16 families based on the differences in the 2^nd^ presenter organ and the mechanics of presentation [10, 24], including (1) enveloping bract presentation: in Araceae, pollen grains from the entire inflorescence are simultaneously exposed on the enveloping bract and adhered to the whole body of the beetle pollinators in the bract, which may promote pollen export [41]; (2) perianth presentation: in *Acrotriche serrulata* (Epacridaceae), pollen grains are presented on the hairs of the terminal petal combs far away from the stigma, which may function to reduce sexual interference [42]; (3) anther filament presentation: in Santalaceae, pollen grains are relocated onto hairs of the anther filaments, which probably functions to lengthen the duration of the male phase [43]; and (4) six types of style presentation (in Marantaceae, Cannaceae, Lobeliaceae, Campanulaceae, Asteraceae, Calyceraceae, Proteaceae, Fabaceae, Myrtaceae, Rubiaceae, Goodeniaceae, Brunoniaceae and Polygalaceae) that may function to enhance precise placement or receipt of the pollen compared to regular anther presentation [24, 25]. However, there has been no previous report of SPP on a labellum staminode through the adherence of aggregated pollen chains. In *Z*. *densissimum*, two floral characters are key for the function of SPP: (1) the close positioning of the anther to the labellum staminode, and (2) the aggregated pollen chains. The former enables pollen to contact the labellum staminode, while the latter promotes more pollen grains adhering to the labellum staminode. The close positioning of anther and labellum staminode occurs in numerous other species of Zingiberaceae (e.g., in genera of *Amomum* and *Etlingera*) without relocation of the pollen on the labellum, so the feature of aggregated pollen chains likely played a more crucial role in the evolution of this unique SPP mechanism in Zingiberaceae.

The occurrence of aggregated pollen chains with SPP has not been reported in nature, although aggregated pollen is widespread in angiosperms [18]. The formation of aggregated pollen chains is correlated with the structures of pollen wall in *Z*. *densissimum*. As shown in our research, the inner layer of the pollen wall (layer III) which reflects fluorescence (Fig 1E and 1F) is supposed to be the callosic inner layer of the intine, while layer II is the pectic outer layer of the intine, according to the palynology of angiosperms [44, 45]. The outer layer (layer I, Fig 1D), which is supposed to be the exine of pollen wall [36], is a structure of spiro-striate sculpturing. The striate sculptures of the exine are slightly straight, with numerous granules between them (Fig 1D), which is consistent with that reported by Liang (1988) [35]. The complicated structure of the exine probably enables pollen grains to adhere to each other, thus forming aggregated pollen chains. The feature of aggregated pollen grains was recorded in *Caulokaempferia coenobialis* (Zingiberaceae). But unlike the pollen chains of *Z*. *densissimum* in which pollen grains are aggregated temporarily through the adherence of pollen grain surface, pollen grains in *C*. *coenobialis* are aggregated permanently through the connection of a structure called pollen connecting- thread [46].

SPP is also found in other two families of Zingiberales: Marantaceae and Cannaceae. Pollen grains in Marantaceae are presented into a spoon-like receptacle at the end of the style. They have only one chance to be removed explosively from the 2^nd^ presenter when a pollinator pushes the spring-trigger of the style [19, 31, 32]. Pollen grains in Cannaceae are presented on the petaloid style before anthesis, which may promote precise placement or receipt of the pollen [24, 33]. However, there is no record of SPP in Zingiberaceae. *Zingiber densissimum* is the first species in which SPP is described in the family. SPP has occurred in the three sister families (Zingiberaceae, Marantaceae and Cannaceae) [47], but mechanisms of SPP among these families are significantly different. Therefore, we can conclude that SPP arose at least three times independently in the order of Zingiberales. Exploring why three different modes of SPP have occurred in three sister families may shed light to the origin and evolution of SPP in angiosperms.

### The functional roles in pollen export and pollination efficiency

In *Z*. *densissimum*, pollen grains are presented both on the anther and on the labellum staminode, enabling pollinators to remove pollen simultaneously from both pollen presenters during a single visit (Fig 3C and 3D). Therefore, this pollen arrangement of SPP promotes pollen export compared with the condition where pollen grains are solely presented on anthers. In addition, pollen grains are deposited on the ventral and the dorsal parts of bees simultaneously, which can enhance the value of the pollinator [22]. This arrangement allows *Z*. *densissimum* to effectively use both a dorsal pollinator and a ventral pollinator, and further promotes pollen export.

The flower of *Z*. *densissimum* has only one fertile anther and lasts for less than one day. All the pollen grains are simultaneously exposed to pollinators after flowers opened. In addition, pollen grains are aggregated into pollen chains, which can enhance pollinator’s capacity to carry pollen besides promoting secondary relocation of the pollen in the labellum [18]. All these features indicate the pollen presentation strategy of *Z*. *densissimum* is simultaneous. For flowers with simultaneous pollen presentation, it is generally more economic to promote the benefit in male function by promoting the amount of pollen export than by enhancing efficiency of pollen deposition [6, 13–16]. That is why the SPP mechanism of *Z*. *densissimum* that promoted pollen export but decreased pollen deposition efficiency can be adaptive.

The two bees were found to have different pollination effectiveness in *Z*. *densissimum*. During a single visit, they removed similar amount of pollen (Fig 3B), but dorsal pollinator (*M*. *hedini*) deposited about twice as many grains on the stigma than ventral pollinator (*A*. *zonata*) (Fig 3A). Although the ventral pollinator had lower effectiveness, it is a valuable pollinator. Pollination is generally dependent on not just the pollinator effectiveness per visit, but also the visitation rate per pollinator individual, and the abundance of pollinator species [48]. In *Z*. *densissimum*, visitation frequency of two bee species fluctuated between 2011 and 2012, with the frequency of ventral pollinator higher than that of dorsal pollinator in 2011 but lower than dorsal pollination bees in 2012. Advantages on reproductive fitness of plants can be achieved through the multi-pollinator system under the condition of temporal variation in pollinator services [48].

Our evaluation on the adaptive significance and the functional roles of SPP was limited to measuring the pollen export and linking pollen export to pollination efficiency, without quantifying the deposition of pollen grains from the 2^nd^ presenter to the stigma. To fully understand the functional roles of this unique secondary pollen presentation mechanism, further studies are needed to link pollen deposition to the pollination efficiency of different pollinators by tracking the deposition efficiency of the grains from the anther and the 2^nd^ presenter, respectively.

The data for this research which were not shown in the manuscript are available in S1 and S2 Tables.

## Supporting Information

S1 Fig A ventral pollinator (*Amegilla zonata*)’s foraging behaviour. The figure shows how pollen grains from the labellum of a flower are deposited on the stigma of other flowers. A ventral pollinator is retreating from a flower after probed nectar, with numerous pollen grains on the tongue, the head and forelegs of the pollinator. These pollen grains are primarily from the labellum. When the pollinator comes to visit other flowers, its forelegs need hold and push the tail-like anther appendage to get the nectar, and the head and forelegs will contact the stigma that is just near the anther appendage. The pollen from the labellum of the previous flower will therefore be deposited on the stigma.
(TIF)

S1 Table The breeding system of *Zingiber densissimum*. (DOC)

S2 Table Pollination efficiency and pollen removal. (DOC)
