# Supplementary figures and images for: A New Secondary Pollen Presentation Mechanism from a Wild Ginger (Zingiber densissimum) and Its Functional Roles in Pollination Process
Source: PLoS One. 2015 Dec 4;10(12):e0143812. doi: 10.1371/journal.pone.0143812 (PMC4670160; doi:10.1371/journal.pone.0143812)

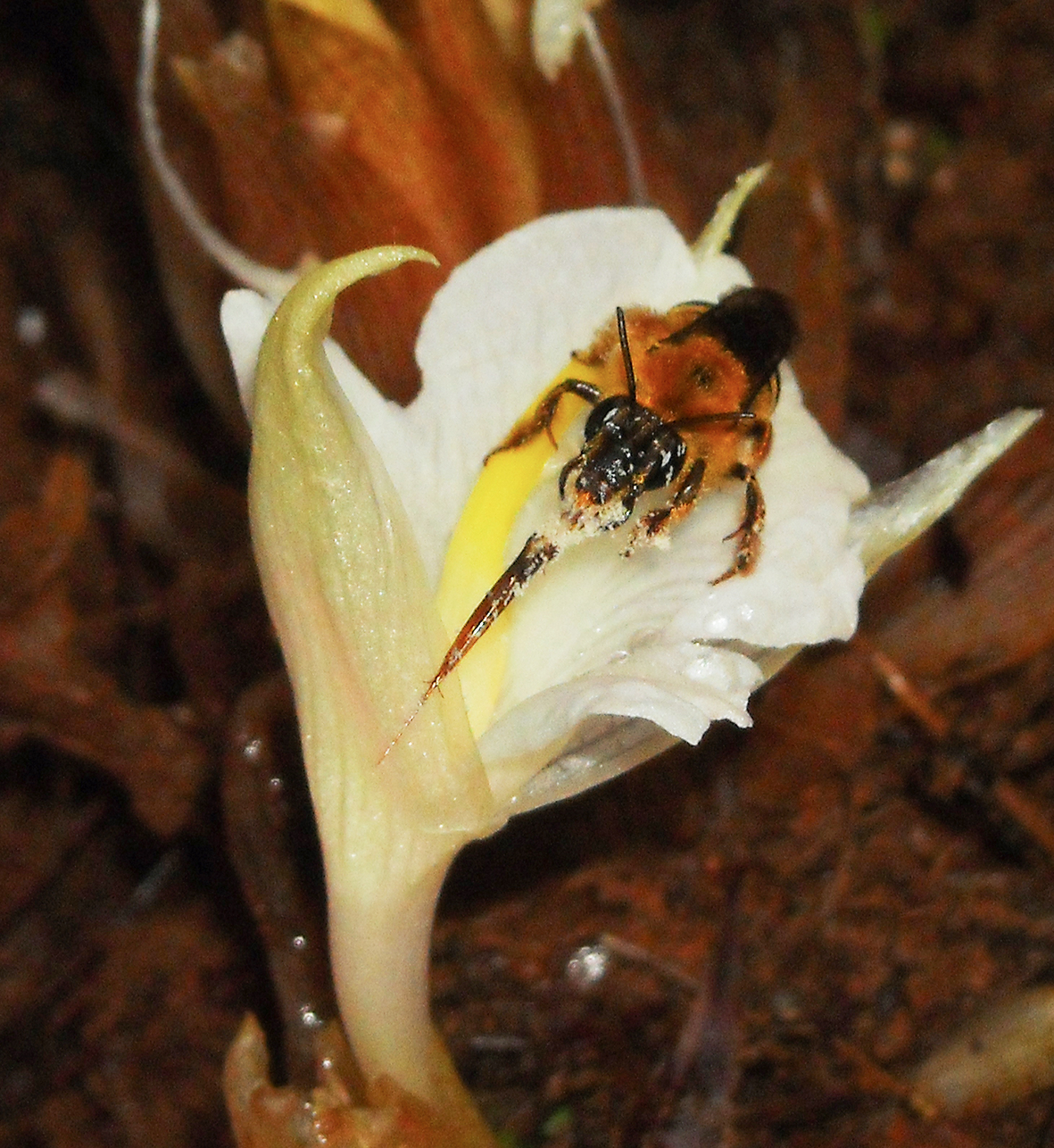

Supplement: S1 Fig — The figure shows how pollen grains from the labellum of a flower are deposited on the stigma of other flowers. A ventral pollinator is retreating from a flower after probed nectar, with numerous pollen grains on the tongue, the head and forelegs of the pollinator. These pollen grains are primarily from the labellum. When the pollinator comes to visit other flowers, its forelegs need hold and push the tail-like anther appendage to get the nectar, and the head and forelegs will contact the stigma that is just near the anther appendage. The pollen from the labellum of the previous flower will therefore be deposited on the stigma. (TIF) [file pone.0143812.s001.tif]
