# Supplementary material for: A New Secondary Pollen Presentation Mechanism from a Wild Ginger (Zingiber densissimum) and Its Functional Roles in Pollination Process
Source: PLoS One. 2015 Dec 4;10(12):e0143812. doi: 10.1371/journal.pone.0143812 (PMC4670160; doi:10.1371/journal.pone.0143812)
Supplement: S1 Table — (DOC) [file pone.0143812.s002.doc]

**S1 Table. Breeding system of *Zingiber densissimum.***

| Year | treatment | Fruit set | Mean seed number ± standard error (sample size) |
| --- | --- | --- | --- |
| 2011 | Emasculated and bagged before anthesis |  | 0 (34) |
|  | Bagged and unmanipulated |  | 0 (50) |
|  | Hand self-pollinated | 88.5% (36) | 12.81 ± 1.08 (32) |
|  | Hand cross-pollinated | 84.4% (45) | 15.94 ± 0.76 (32) |
| 2012 | Hand self-pollinated | 74.2% (31) | 9.65± 0.86 (23) |
|  | Hand cross-pollinated | 76.7 % (30) | 12.74± 0.68(23) |
