# Supplementary material for: A New Secondary Pollen Presentation Mechanism from a Wild Ginger (Zingiber densissimum) and Its Functional Roles in Pollination Process
Source: PLoS One. 2015 Dec 4;10(12):e0143812. doi: 10.1371/journal.pone.0143812 (PMC4670160; doi:10.1371/journal.pone.0143812)
Supplement: S2 Table — (DOC) [file pone.0143812.s003.doc]

**S2 Table. Pollination efficiency and pollen removal.** Seed production and pollen left after a single visit by pollinator (mean + s.e., with sample size in brackets)

| Pollinator type | Seed production | Total pollen grains | Pollen on the anther | Pollen on the labellum | Remark |
| --- | --- | --- | --- | --- | --- |
| Control | 0 | 8782 ± 162 (30) | 5394 ± 131 (30) | 3388 ± 99 (30) | Control : flowers are not visited by pollinator |
| Dorsal pollination | 5.13±0.97 (40) | 6659± 198 (30) | 3775± 190 (30) | 2284 ± 156 (30) |
| Ventral pollination | 2.51±0.51(43) | 6475 ± 110 (30) | 4499 ± 72 (30) | 1975 ± 45 (30) |
